# LRP1 as a potential diagnostic and immunomodulatory target in endometriosis: evidence from multi-omics and single-cell analyses

**DOI:** 10.3389/fimmu.2026.1735221

**Published:** 2026-04-15

**Authors:** Chengmao Xie, Yong Liu

**Affiliations:** Department of Gynecology, Beijing Obstetrics and Gynecology Hospital, Capital Medical University. Beijing Maternal and Child Health Care Hospital, Beijing, China

**Keywords:** biomarker, cell-cell communication, endometriosis, LRP1, machine learning, mendelian randomization analysis

## Abstract

Endometriosis (EMS) is a common gynecological disease that seriously affects women’s health and quality of life. However, the detailed dynamic cellular and molecular mechanisms underlying EMS pathogenesis remain largely unknown. This study establishes a novel diagnostic model to distinguish ectopic endometrium (EC) from eutopic endometrium (EU) samples. This study elucidated the critical role of low-density lipoprotein receptor-related protein 1 (LRP1) in EMS pathogenesis through integrated multi-omics analyses. Our comprehensive approach began with merging three GEO datasets (GSE7305, GSE11691, GSE25628), identifying 1,404 differentially expressed genes (DEGs) and 489 co-expressed genes with the brown module using weighted gene co-expression network analysis (WGCNA). A machine learning algorithm identified 30 hub genes, among which LRP1 exhibited the highest diagnostic performance. Immune profiling revealed a strong positive correlation between LRP1 and M2 macrophage infiltration (r=0.62, p<0.001), while Mendelian randomization analysis confirmed a causal relationship (OR = 1.35, 95%CI:1.13-1.61, p=0.001). Single-cell RNA sequencing demonstrated LRP1 is predominantly expressed in fibroblasts and monocytes, orchestrating cell-cell communication through the MIF signaling pathway, particularly through CD74/CXCR4 interactions. Experimental validation confirmed significantly elevated LRP1 expression in ectopic lesions at both mRNA (p<0.01) and protein levels. Mechanistically, LRP1 may promote the progression of EMS by enhancing cell migration, invasion, and proliferation through the MIF signaling pathway. Collectively, these findings identified LRP1 as a central regulator of EMS progression through immunomodulation and intercellular crosstalk, offering novel diagnostic and therapeutic potential.

## 1 Introduction

Endometriosis (EMS) is an estrogen-dependent disease commonly seen in women of childbearing age, with an incidence rate of approximately 5-10%, and more than 176 million women worldwide suffer from EMS (1). Women with EMS often experience infertility, pelvic pain, dysmenorrhea, and painful intercourse, and approximately 71-87% of women with pelvic pain and 20-50% of infertile women could be observed in the presence of EMS (2). EMS mainly affects the pelvic cavity, but this can also occur outside the pelvic cavity or even in thoracic organs (3). Although EMS is a benign disease in histology, it has malignant biological potential, such as implantation, infiltration, malignancy, and recurrence, and is highly invasive (4). Although present medical treatment can alleviate symptoms in up to 50-80% of cases, at least 20% of patients show no medical response due to the complex and unknown mechanism of EMS pathogenesis (5–7).

The onset of EMS may be closely associated with the age of menarche, menstrual cycle, menstrual flow, smoking habits, and other lifestyle factors in women. EMS can impair the function of various cell types, including macrophages, endothelial cells, keratinocytes, and epidermal cells (8). However, there is no consensus on the specific EMS pathogenesis and mechanisms of disease progression (9). At present, the diagnostic methods for EMS lack specificity, and there are no specific serological indicators. Although some EMS patients may have elevated CA125 levels, this happens more commonly in patients with severe EMS, combined with ruptured ovarian endometriosis cysts or adenomyosis. In addition, CA125 does not show diagnostic value for early EMS patients and lacks specificity and sensitivity in diagnosing EMS (10). Low-density lipoprotein (LDL) receptor-related protein 1 (LRP1) is a large endocytic receptor that is broadly expressed across various tissues. As a member of the LDL receptor family, LRP1 plays diverse roles in various biological processes, including lipoprotein metabolism, protease degradation, lysosomal enzyme activation, and cellular entry of bacterial toxins and viruses (11). Emerging evidence suggests that LRP1 participates in the pathological process of EMS through multiple mechanisms. It regulates macrophage polarization, promoting M2 phenotype formation, which is highly consistent with the immunosuppressive microenvironment characteristic of EMS lesions (12). Additionally, LRP1 participates in extracellular matrix remodeling and affects tissue invasion by endocytic proteases such as MMP9, key factors in the invasive behavior of EMS. Moreover, LRP1-mediated lipid metabolism abnormalities are closely linked to the changes in blood lipids in EMS patients. Given its pivotal role in inflammation, matrix remodeling, and metabolic reprogramming, LRP1 represents a promising target for elucidating the pathogenesis and developing therapeutic strategies of EMS (13, 14). Although the aforementioned evidence suggests that LRP1 may be involved in EMS through multiple pathways, including immune regulation, stromal remodeling, and metabolism, its systemic role in the initiation and development of EMS remains unclear, particularly the mechanism by which it functions as a hub for intercellular communication within the immune microenvironment. Furthermore, validation using a large-scale omics dataset is still lacking, and it remains uncertain whether LRP1 can serve as a reliable diagnostic biomarker for EMS.

To enhance the understanding of the pathogenesis and molecular mechanism of EMS and to identify novel targets for clinical diagnosis and treatment, the discovery of new biomarkers became crucial. In recent years, machine learning and bioinformatics technologies have been widely applied to high-throughput data analysis, enabling the identification of key disease-related genes and providing new insights into clinical diagnosis and treatment (15). Furthermore, single-cell RNA sequencing (scRNA-seq), an emerging technology, allows for the identification of cell types and subtypes related to various diseases, as well as exploration of gene expression dynamics and cell development trajectories across heterogeneous populations. The application of these advanced technologies provides powerful tools for an in-depth understanding of the complex molecular mechanism of EMS (8, 16, 17). Therefore, this study aims to systematically identify key pathogenic genes of EMS by integrating multi-cohort transcriptome data, machine learning approaches, Mendelian randomization-based causal inference, and single-cell RNA sequencing analysis. We further focus on exploring the role of LRP1 in regulating the immune microenvironment of EMS and evaluating its potential as a diagnostic marker.

The present study obtained the tissue RNA expression profiling data of EU and EC samples obtained from the Gene Expression Omnibus (GEO) database. Then, an integrated and comprehensive bioinformatic analysis combined with laboratory research work was performed to identify the potential pathogenic genes of EMS. The present study results revealed that low-density LRP1 plays a crucial role in the pathogenesis of EMS by affecting cell-cell communication via the ligand-receptor interaction, particularly its interactions with other immune cells. The findings may provide new insights into the pathological mechanisms of EMS linked to the immune environment of EMS, and may help identify innovative early diagnostic biomarkers and therapeutic targets for patients with EMS.

## 2 Materials and methods

### 2.1 Data acquisition and processing

Five EMS-related datasets with RNA expression profiles (GSE5108 (n=22), GSE7305 (n=20), GSE11691 (n=18), GSE25628 (n=16), and GSE37837 (n=36)) were downloaded from the GEO database (https://www.ncbi.nlm.nih.gov/geo/), which is a public database that contains high-throughput microarrays and next-generation sequence datasets (18). In addition, a single-cell expression dataset of eight EU and eight EC endometrium tissues was obtained from GSE179640. The robust multi-array averaging (RMA) algorithm implemented in the “affy” software package was employed to preprocess the collected data (19).

### 2.2 Differentially expressed gene analysis

Since the GSE7305, GSE11691, and GSE25628 datasets all contain paired ectopic (EC) and eutopic (EU) endometrial tissue samples from patients at various stages of EMS, they were used to analyze the differential gene expression between EC and EU tissues. The three files were merged into one file, and the “sva” R package was used to normalize the expression data of the combined dataset. To assess the effectiveness of batch correction, principal component analysis (PCA) on both pre- and post-correction data was performed. Finally, a normalized gene expression matrix file that contained the data obtained from the combined dataset (including 28 EU and 26 EC endometrium, training dataset) was obtained for DEG analysis. The DEGs were identified from the EC and EU endometrial tissues using the “limma” package, with the |Log_2_FC| value of >1.5 and *p-value* of <0.05 as thresholds. To further explore the functional characteristics of candidate genes in endometriosis (EMS), we classified them into down-regulated genes and up-regulated genes based on their expression patterns. Subsequently, the R package clusterProfiler (version 4.0) was used to conduct Gene Ontology (GO) and Kyoto Encyclopedia of Genes and Genomes (KEGG) pathway enrichment analyses on these two groups of genes, respectively (20).

### 2.3 Weighted gene co-expression network analysis (WGCNA) collaborative expression network construction

Initially, principal component analysis (PCA) was conducted using the whole gene and DEG list. Then, WGCNA was performed to create a co-expression network of all genes in the EC and EU endometrial samples. Genes with the top 25% variance were filtered for further analysis. Then, 28 EC and 26 EU samples were separately subjected to WGCNA. Afterwards, the samples were used to calculate the Pearson correlation matrix and topological overlap metric (TOM) matrix, as previously described (21).

### 2.4 Identification of EMS-related DEGs

The list of genes in the brown module obtained from WGCNA was cross-referenced with the list of DEGs. Cross genes were designated as candidate hub genes and used for the subsequent analysis.

### 2.5 Enrichment of DEGs functions and pathways

Several software packages were employed to characterize the function and pathways of DEGs in the EC and EU samples. The Gene Ontology (GO) database, which defines the biological processes (BP), cellular components (CC), and molecular functions (MF), has been widely used to explain genome alterations (22). The KEGG database provides genomic and functional information for gene function analysis (23–25). The Database for Annotation, Visualization, and Integrated Discovery (DAVID, https://davidbioinformatics.nih.gov/) is an online tool that provides functional annotations and visualizations to gain insights into the biological themes enriched in genome-scale studies (26, 27). The present study performed the GO analysis using DAVID and enriched the KEGG pathway of DEGs. A *p-value* of <0.05 was considered statistically significant, and the results were visualized using ggplot2 in the R package.

### 2.6 Integrating machine learning algorithms to construct potential biomarkers for EMS

A computational pipeline incorporating ten machine learning algorithms was applied to conduct a comprehensive analysis of the candidate hub genes for the purpose of identifying robust EMS-related hub genes. Detailed specifications of these ten algorithms have been described in a previous study (28). The construction of hub genes related to EMS was performed through the following four steps: (1) fitting the prediction model of the GEO dataset with 101 algorithm combinations; (2) all algorithm combinations were executed in the GEO queue; (3) the C-index of all queues was calculated; (4) the running process of the R script (https://github.com/Zaoqu-Liu/IRLS) was executed according to previous studies (28, 29).

### 2.7 Diagnostic value of hub genes to predict EMS

Receiver operating characteristic (ROC) analysis was performed to calculate the area under the curve (AUC) for the three datasets (GSE5108, GSE37837, and training dataset) using the “pROC” feature of the R software (version 4.3.3). AUC>0.9, 0.7<AUC ≤ 0.9, and 0.5<AUC≥0.7 indicated the high, moderate, and low diagnostic values, respectively.

### 2.8 Confusion matrix for binary classification

Each column of the confusion matrix represents the actual sample situation of a class, and each row represents the predicted situation of a class. The number of positive and negative samples was referred to as (P) and (N), respectively, and the number of correctly predicted positive and negative samples was referred to as true positive (TP) and false positive (FP), respectively. The number of correctly predicted negative and positive samples was referred to as false negative (FN) and true negative (TN), respectively. The evaluation items included accuracy, precision, recall, and F1-score. The calculation formula was, as follows (worst value = 0, best value = 1) (30):


$$\text{Accuracy}=\frac{\text{TP}+\text{TN}}{\text{TP}+\text{TN}+\text{FP}+\text{FN}}\;\;\;\;\;\text{Precision}=\frac{\text{TP}}{\text{TP}+\text{FP }}$$

$$\text{Recall}=\frac{\text{TP}}{\text{TP}+\text{FN}}\;\;\;\;\;\text{F}1-\text{Score}=\frac{2*\text{TP}}{2*\text{TP}+\text{FP}+\text{FN}}\;$$

### 2.9 Visualization, correlation, ROC curve, and protein-protein interaction (PPI) network analysis of hub genes

The visualization of the expression of hub genes selected by the integrated machine learning models (Enet (alpha=0.3)) in each group was analyzed using “ggplot2” and “aggregate”, with the following filtering conditions: logFCfilter = 0.585 and adj.P.Val. Filter = 0.05. Correlation analysis was conducted for the hub genes selected by the integrated machine learning models using the “ggcorrplot” and “PerformanceAnalytics” packages. The drawing of the ROC curve and calculation of the AUC were performed with reference to step 2.7. The functionally similar genes of hub genes were predicted through the GeneMANIA website (http://genemania.org), and the PPI network was constructed among these (31). The present study used the GeneMANIA tool to conduct the functional enrichment analysis of DEGs and hub genes (32).

### 2.10 Immune infiltration and immuneCor analysis

CIBERSORT is a deconvolution tool to infer cell type abundances based on the gene expression data (33). The present study used the CIBERSORT software to analyze the proportion of 22 types of immune cell infiltration in each tissue from the training set (Samples with p > 0.05 were excluded). For each sample, the sum of all evaluated immune cell types was equal to 1 (34). Then, the linkET package was used to explore and visualize the correlation between hub genes and immune cell activities. The correlation between hub genes and the levels of infiltrating immune cells was explored using the “Spearman” package. Subsequently, the “ggplot2”, “reshape2”, “ggpubr”, and “ggExtra” packages were used to visualize the correlation between characteristic genes and immune cell infiltration levels. To further validate the expression differences of the 30 candidate genes in EMS, we analyzed their expression profiles in the validation datasets GSE37837 and GSE5108. The Wilcoxon rank-sum test was used to compare the expression levels of candidate genes between the disease group (EC) and the normal group (EU), with a significance threshold set at p<0.05. Boxplots illustrating the expression differences of candidate genes between EC and EU samples were generated using the ggpubr package (version 0.6.0) in R (35).

### 2.11 LRP1-related differential gene analysis and functional enrichment analysis

Based on the median expression level of LRP1 in the integrated dataset, 26 disease samples were divided into LRP1-low (n=13) and 13 LRP1-high (n=13) groups. Differential gene analysis between the two groups was performed using the R package Limma (version 3.56.2), with thresholds set at |log_2_FC| > 0.5 and p value < 0.05 to identify LRP1-associated DEGs (36). To explore the biological processes and signaling pathways potentially involved by these DEGs, GO (including Cellular Components, CC; Biological Processes, BP; and Molecular Functions, MF) and KEGG pathway enrichment analyses were conducted in the GSE7305, GSE11691, and GSE25628 datasets using the clusterProfiler package (4.10.1), with a significance defined as p value < 0.05 (20). Enrichment results were ranked by gene count, and in cases of ties, the pathway with the smaller p value was prioritized. The top five pathways were selected for visualization.

### 2.12 The eQTL analysis of exposed data and the determination of outcome data

In order to identify the association between genetic variations and gene expression, eQTL analysis was performed using the genotype and transcriptome data obtained from the different cohort samples. The source of the eQTL data used for the present study was the Genome Wide Association Study (GWAS) catalog website (https://gwas.mrcieu.ac.uk/). The GWAS data of EMS was obtained from the GEO database (https://www.finngen.fi/en), after obtaining R10_manifest, filtering the table with EMS as the keyword, and downloading relevant data from the corresponding link. The R package “TwoSampleMR” was used to characterize the correlated single-nucleotide polymorphisms (SNPs). The linkage imbalance parameter was set to r^2^ <0.001, and the aggregation distance was 10,000 kb. SNPs with weak associations or insufficient explanation for phenotype variance were excluded, with an F-test value of >10. All GWAS summary statistical data cited in the present study are publicly accessible and downloadable. Based on the initial analysis, the present study obtained ethical approval.

### 2.13 Mendelian randomization analysis

To conduct the MR analysis, three key assumptions needed to be met: (1) the selected SNPs should be significantly correlated to the exposure level; (2) the SNPs should be independent of potential confounding factors; (3) the SNPs should be specifically associated with outcome risk. The “TwoSampleMR” package was used for the MR analysis in the present study. The inverse variance weighted (IVW) approach was used to determine the correlation between distinct genes and EMS. Furthermore, an additional sensitivity analysis was performed using the MR Egger, simple mode, weighted median, and weighted mode approaches. The triple criteria method was used to identify disease-related genes: (1) genes were selected with a *p-value* of <0.05 using the IVW method; (2) genes were further processed based on the consistency of the MR analysis result (ratio); (3) genes presenting pleiotropy signs with a *p-value* of <0.05 were excluded from the analysis.

Subsequently, co-expressed genes between these EMS-related genes and DEGs were identified through a cross-identification approach. Then, MR analysis was separately performed for all intersecting genes to determine the causal relationship with the disease. The robustness and reliability of the results were evaluated using multiple analyses, which included heterogeneity and pleiotropy testing, as well as omission sensitivity analysis.

### 2.14 Gene set enrichment analysis and gene set variation analysis enrichment analyses

GSEA is a tool for exploring the functional role and pathways related to diagnostic genes. GSEA enrichment analysis was used to analyze the activity levels of relevant functions or pathways related to critical gene sets.

The Gene Set Variation Analysis (GSVA) algorithm was used to comprehensively score the core genes and assess the potential functional changes under different expression conditions.

### 2.15 The scRNA-seq data and intercellular communication analysis

The present study utilized the raw gene expression matrix obtained from the GSE179640 dataset, which contains the scRNA-seq data from eight EMS patients. The objective of this study was to analyze the immune cell composition and molecular characteristics of the EMS tissues’ microenvironment. The Seurat R package (version 4.0.4) was used for the quality control (QC) process. The filtering criteria were as follows: (1) the number of genes exceeded 300; (2) the cell number was between 1,000 and 20,000; (3) the mitochondrial gene content was <12.5%, the ribosomal gene content was >0.3%, and the red blood cell gene context <3%. After QC, the cell populations were annotated based on the marker genes and visualized using t-distributed stochastic neighbor embedding (t-SNE) for dimensionality reduction.

The CellChat tool was employed to characterize the intercellular communication networks from the scRNA-seq data. Then, ligand-receptor interaction analysis was employed to identify the cell-cell communication linked to the central genes in the EMS environment.

### 2.16 Key cell signaling and trajectory analysis

To explore intercellular communication involving fibroblasts, the “netVisual_bubble” function from the CellChat package (2.2.0) was used to visualize the signal intensities of ligand-receptor pairs related to the output and input signals of fibroblasts. Fibroblasts were further subjected to dimensionality reduction and clustering (dim=3, resolution =0.4), resulting in the classification of two fibroblast subpopulations: fibroblasts 1 and fibroblasts 2 (37). The differentiation trajectory of fibroblasts along with the dynamic expression pattern of LRP1 was analyzed using the single-chip analysis software Monocle package (2.30.1), with results visualized through DDRTree plots (37). Cell type abundance was illustrated using ggplot2, and the expression of LRP1 across cell types was shown using the dot plot and feature plot generated by Seurat.

### 2.17 Clinical samples

The present study selected 30 EMS patients who underwent hysteroscopy combined with laparoscopic surgery at Beijing Obstetrics and Gynecology Hospital from January 2021 to December 2023, and the basic clinical information of those 30 enrolled patients was provided in **Supplementary File 1**. During the surgery, some EU and EC tissues were collected. Some of the samples were snap-frozen in liquid nitrogen and stored at -80 °C, while the other samples were quickly soaked in formalin solution. The present study obtained informed consent from the patients and their families and was approved by the hospital ethics committee.

### 2.18 Cell culture and transfection

Human endometrial stromal cells (12Z) were purchased from Procell (Wuhan, China) and cultured using DMEM/F12 medium (Thermo Fisher Scientific) supplemented with 10% fetal bovine serum (FBS) and 1% penicillin streptomycin in a humidified incubator (Heracell™ VIOS 160i, Thermo Fisher Scientific) at 37°C with 5% CO_2_. All cell handling procedures were performed in a Class II A2 Biosafety Cabinet (ESCO). Cells were used for experiments at 70–90% confluence. At approximately 70% confluence, transfections were performed using Lipofectamine 3000 (L30000008, Thermo Fisher Scientific) according to the manufacturer’s instructions. The transfection groups were as follows: LRP1 knockdown (50 nM LRP1 siRNA,sc-43956, Santa Cruz Biotechnology); Negative control (50 nM negative control siRNA,sc-37007, Santa Cruz Biotechnology); MIF knockdown (50 nM siRNA-MIF,sc-35910, Santa Cruz Biotechnology); LRP1 and MIF double knockdown (50 nM siRNA-LRP1 and 50 nM siRNA-MIF with a final concentration of 50 nM for each siRNA); Cells were harvested 48 hours after transfection, and knockdown or overexpression efficiency of LRP1 and MIF was confirmed by Western Blot analysis.

### 2.19 Immunohistochemistry

Immunohistochemistry was performed for the EU and EC samples. Briefly, the EC and EU samples were fixed in formalin and embedded in paraffin. Then, 4 μm thick sections were prepared and heated at 60 °C for 10 minutes, followed by dewaxing, rehydration, and washing in phosphate-buffered saline (PBS, pH 7.4). Then, the sections were incubated in 3% H_2_O_2_ for 10 minutes to inactivate the endogenous peroxidase and washed in PBS for 3×5 minutes. Afterwards, the glass slides were subjected to antigen retrieval in sodium citrate buffer (pH 6.0) for 15 minutes (3×5 minutes) in an 850 W microwave oven, cooled at room temperature for two hours, and washed in PBS for 3×5 minutes. Next, the sections were blocked by preincubating with normal goat serum (1:10) for 30 minutes, incubated overnight with the rabbit monoclonal anti-human LRP1 antibody (Ab92544, 1:200; Abcam, Cambridge, UK) at 4 °C, and washed in PBS for five minutes. Then, the sections were incubated with biotinylated goat anti-rabbit antibody (1:500) at room temperature for 30 minutes and washed in PBS for 3×5 minutes. Afterwards, DAB and hematoxylin staining were performed, and the staining signals were observed under a microscope. The isotype control on each sample was performed by replacing the anti-LRP1 antibody with mouse serum. In order to compare the difference in LRP1 protein levels between the EC and EU samples, the Image Pro Plus software (6.0) was used to measure the integrated optical density values of all samples.

### 2.20 RNA isolation and quantitative real-time polymerase chain reaction

Total RNA was isolated from the EU and EC endometrial samples using TRIzol reagent (Thermo Fisher Scientific, Waltham, Massachusetts, USA). Then, the concentration of RNA was determined using a spectrophotometer. Afterwards, 2 μg of total RNA from each sample was reverse transcribed in a 20 µl reaction system using a gradient cycler (Thermo Fisher Scientific, Waltham, Massachusetts, USA). The cDNA synthesis was performed at 25 °C for 10 minutes and at 37 °C for 120 minutes. Then, this was incubated at 85 °C for five minutes, and at 4 °C for 10 minutes. Afterwards, qRT-PCR was performed to determine the LRP1 expression using LightCycler DNA Master SYBR Green (Roche Diagnostics, Germany). The sense and antisense primers for LRP1 were 5’-TCAACTACAACCGGACCGTG-3’ and 5’-TCCACA CCTCGGATCTCCAT-3’, respectively. The length of the amplicon was 127 bases, and β-actin was used to normalize the expression data with the sense and antisense primer 5’-CATGTACGTTGCTATCCAGGC-3’ and antisense primer 5’-CTCCTTAATGTCACGCAC GAT-3’. The polymerase chain reaction (PCR) reaction consisted of 3 mM of MgCl_2_, 0.4 μM of forward and reverse primers, and 1 μL of LightCycler DNA Master SYBR Green I (10-fold concentrate, Roche). The cycling conditions included an initial denaturation step at 95 °C for 75 seconds, followed by 40 cycles, which included denaturation at 95 °C for 15 seconds, annealing at 62 °C for 10 seconds, and elongation at 72 °C for 25 seconds. In order to remove non-specific signals before SYBR Green I quantification, a condition that involved high-temperature fluorescence collection points was added to each amplification cycle, namely, LRP1 at 80 °C for three seconds, and β-actin at 85 °C. After the last cycle, melting curve analysis was performed on the amplified product to check the integrity of the amplification. The data was analyzed using the LightCycler relative quantification software and the second derivative maximum method. The LRP1 values were expressed relative to the β-actin expression. The amplification of specific LRP1 mRNA was assessed by agarose gel electrophoresis to monitor the amplicon size.

### 2.21 Protein preparation and immunoblot analysis

Immunoblot analysis was performed for the EU, EC endometrial tissues and cells. The samples were crushed in liquid nitrogen, and the membrane proteins were extracted according to the manufacturer’s instructions (Thermo Fisher Scientific, Waltham, Massachusetts, USA). Cells were lysed using RIPA buffer (Thermo Fisher Scientific, Waltham, MA, USA). The concentration of protein was quantified using the Quick Start Bradford protein assay (Bio-Rad, Hercules, CA, USA). Sodium dodecyl-sulfate polyacrylamide gel electrophoresis (SDS-PAGE) was carried out on 7% glyceryl triacetate gel with approximately 20 μg of protein samples and operated at 120 V for two hours. In order to maintain the integrity of the LRP1, the samples were not heated before the electrophoresis. After electrophoresis, the proteins were transferred onto a 300 mA Immobilon NC transfer membrane for 90 minutes. Then, the membrane was blocked with 5% skim milk powder in triple-buffered saline that contained 0.1% Tween 20 (TBST) for two hours. Next, the membrane was incubated overnight with the rabbit monoclonal anti-human LRP1 (Abcam, ab92544), MIF (Abcam, ab187064), CD74 (Abcam, ab108393), CXCR4 (Abcam, ab181020), CD44 (Abcam, ab243894), or β-actin (Abcam, ab6276) antibody (1:1,000 in TBST) at 4 °C. Then, the membrane was washed with TBST for 3×10 minutes. Afterwards, the membrane was incubated with the horseradish peroxidase (HRP) secondary antibody (1:5,000 in TBST) that binds to mouse anti-rabbit IgG at room temperature for 45 minutes. Subsequently, the membrane was washed with TBST for 3×10 minutes. The protein band signals were detected by chemiluminescence (ECL System, Amersham), and β-actin was used as the internal loading control.

### 2.22 Transwell invasion assay

Transwell chambers (8 µm pore size, 24-well plate, Corning) were precoated with Matrigel (Corning, 356234) at a 1:8 dilution. Treated cells (5×10^4^ cells/chamber) were suspended in serum-free DMEM/F12 and seeded in the upper chamber. The lower chamber was filled with DMEM/F12 containing 10% FBS to serve as a chemoattractant. After incubation for 24 hours at 37 °C, cells that had migrated to the lower surface of the membrane were fixed, stained with crystal violet, and counted under an inverted microscope (Eclipse Ts2R, Nikon) in five randomly selected fields per chamber.

### 2.23 Wound healing assay

Cells were seeded in 6-well plates and grown to 90% confluence. A scratch was created using a 200 μL pipette tip, and detached cells were removed by washing with PBS. Images of the scratch were captured at 0 and 24 hours using an inverted microscope (Eclipse Ts2R, Nikon). Cell migration was quantified by measuring the reduction in scratch width over time using ImageJ software.

### 2.24 Cell viability assay

A total of 3× 10^4^ cells in 100 μL of culture medium were seeded into 96-well plates and cultured for 24 h. Then, 10 μL of Cell Counting Kit-8 (CCK-8) reagent (MA0218-2, Meilunbio, China) was added to each well. After an additional two-hour incubation in a humidified atmosphere at 37 °C with 5% CO2, absorbance was measured at 450 nm using a spectrophotometer. Each condition was tested in triplicate.

### 2.25 Statistical analysis

All statistical analyses were performed using the R 4.3.3 software. The correlation between two continuous variables was evaluated using the Pearson correlation coefficient. Categorical variables and continuous variables were compared by chi-square test and Wilcoxon rank sum test/t-test, respectively. All statistical tests are two-sided tests. A *p-value* of <0.05 was considered statistically significant.

## 3 Results

### 3.1 Data overview and preprocessing

**Figure 1:**
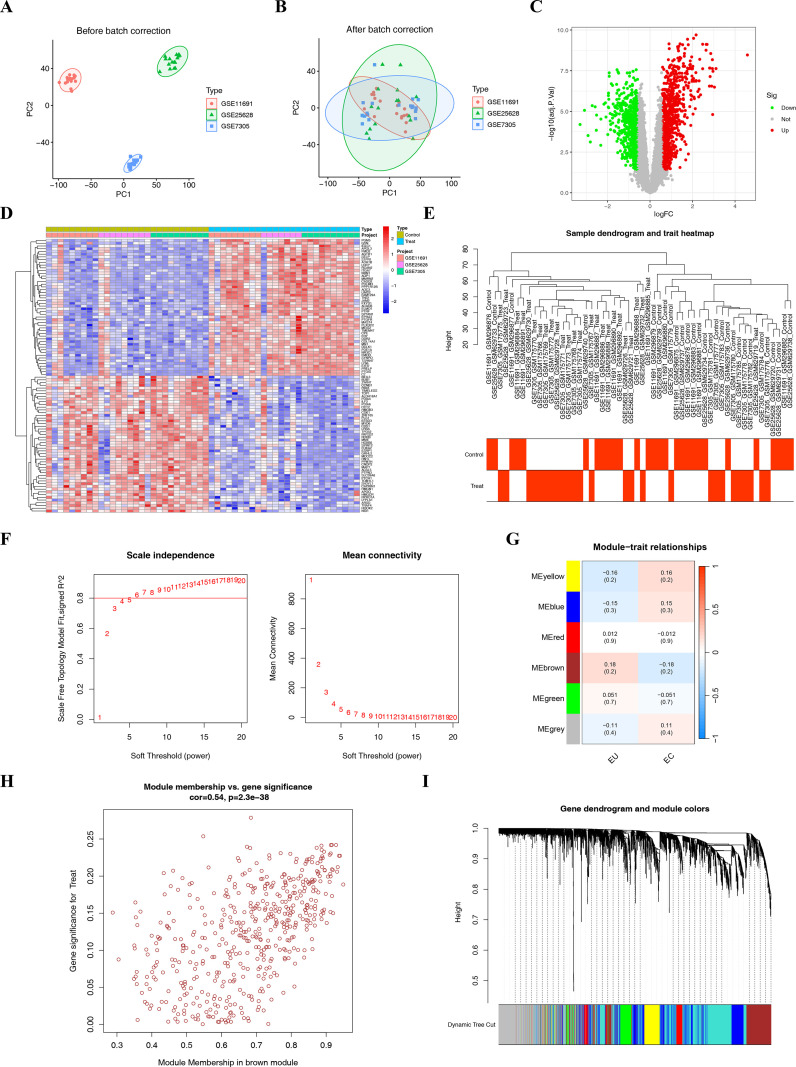
Identification of DEGs and module genes. **(A, B)** Integration of the three GEO datasets. **(A)** Before batch correction; **(B)** After batch correction; **(C)** Volcano plot of DEGs for the EU and EC samples, red represents up-regulated genes, green represents down-regulated genes, and gray represents non-significant genes; **(D)** Heatmap of the top 50 up- and down-DEGs; **(E)** Abnormal sample detection. All gene sets of the samples are included in the dendrogram; **(F)** The hierarchical clustering of gene modules identified by WGCNA was used to generate the tree diagram, and the colors of the modules below represent the gene clustering; **(G)** Soft threshold screening; **(H)** Module-disease correlations; **(I)** The brown gene module exhibited the highest correlation with EMS, with a correlation coefficient of 0.54, which includes 489 genes.

Three EMS microarray datasets obtained from the GEO database were integrated to create a training dataset, which included a total of 54 participants. **Table 1** provides the detailed information on the included datasets. Then, R version 4.3.3 was used to correct and merge the expression values of each gene in its respective datasets, and batch effects were eliminated. As shown in **Figure 1A**, the batch processing effect was evident in the three EMS-related gene datasets. After correction, all samples in the combined dataset achieved acceptable homogeneity with the evidence of the PCA analysis (**Figure 1B**).

**Table 1:** Characteristics of the three GEO datasets.

GSE ID	Samples	Tissues	Platform	Experiment type	Last update date
GSE7305	10 EU and 10 EC	Endometrial tissue	GPL570	Array	March 25, 2019
GSE25628	9 EU and 7 EC	Endometrial tissue	GPL571	Array	December 06, 2018
GSE11691	9 EU and 9 EC	Endometrial tissue	GPL96	Array	August 10, 2018

### 3.2 Identification of 1,404 DEGs and 489 module genes

In the training dataset, 1,404 DEGs were identified from the EU and EC samples. Among these, 802 and 602 genes were upregulated and downregulated, respectively (**Figure 1C**). The top 50 most significantly up- and down-DEGs are presented in **Figure 1D**. In the weighted gene co-expression network clustering analysis, all gene sets of the samples were included in the tree diagram (without outliers) (**Figure 1E**). In order to construct a scale-free network, the soft threshold was set to 6 (R2 = 0.8, **Figure 1F**), and six modules were ultimately identified (**Figures 1G, H**). According to the screening results of the WGCNA, the brown module with the largest |Cor| that contained 489 genes was selected for further analysis (**Figure 1I**).

### 3.3 Functional enrichment analysis revealed that LRP1 is involved in immune regulation and the cell cycle pathway

**Figure 2:**
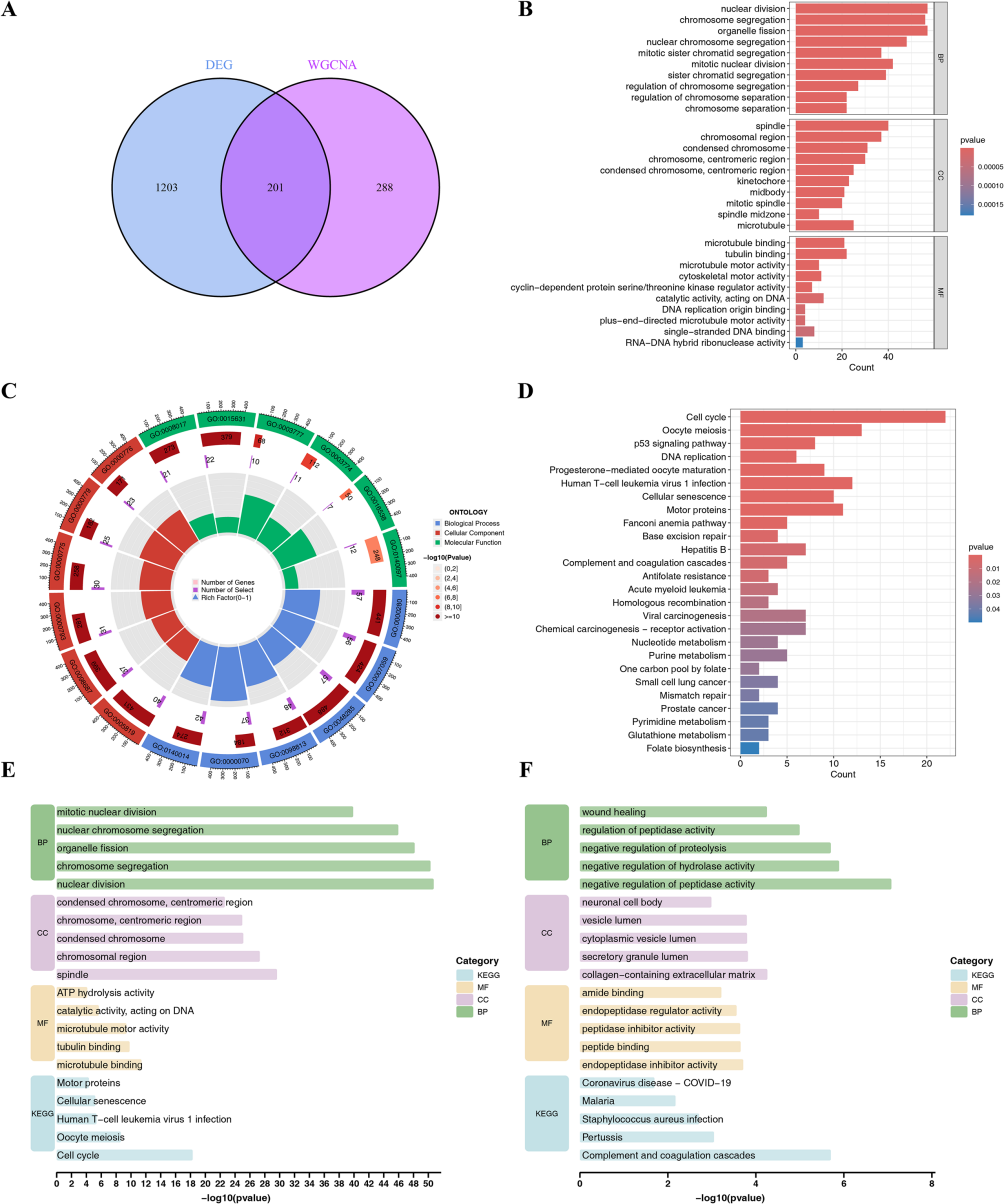
Screening and functional enrichment analysis of candidate genes. **(A)** The Venn diagram presents the 201 common genes in the DEGs and genes from the brown co-expression module associated to EMS; **(B)** The Gene Ontology (GO) enrichment analysis of the 201 crossed genes, which were classified into the following categories: biological processes (BP), cellular components (CC), and molecular functions (MF); **(C)** The GO circle illustrates the scatter map of logFC for the specified genes; **(D)** Bar plot of the KEGG enrichment results of candidate genes; **(E)** Enrichment results of 169 down-regulated genes; **(F)** Enrichment results of 32 upregulated genes.

**Figure 3:**
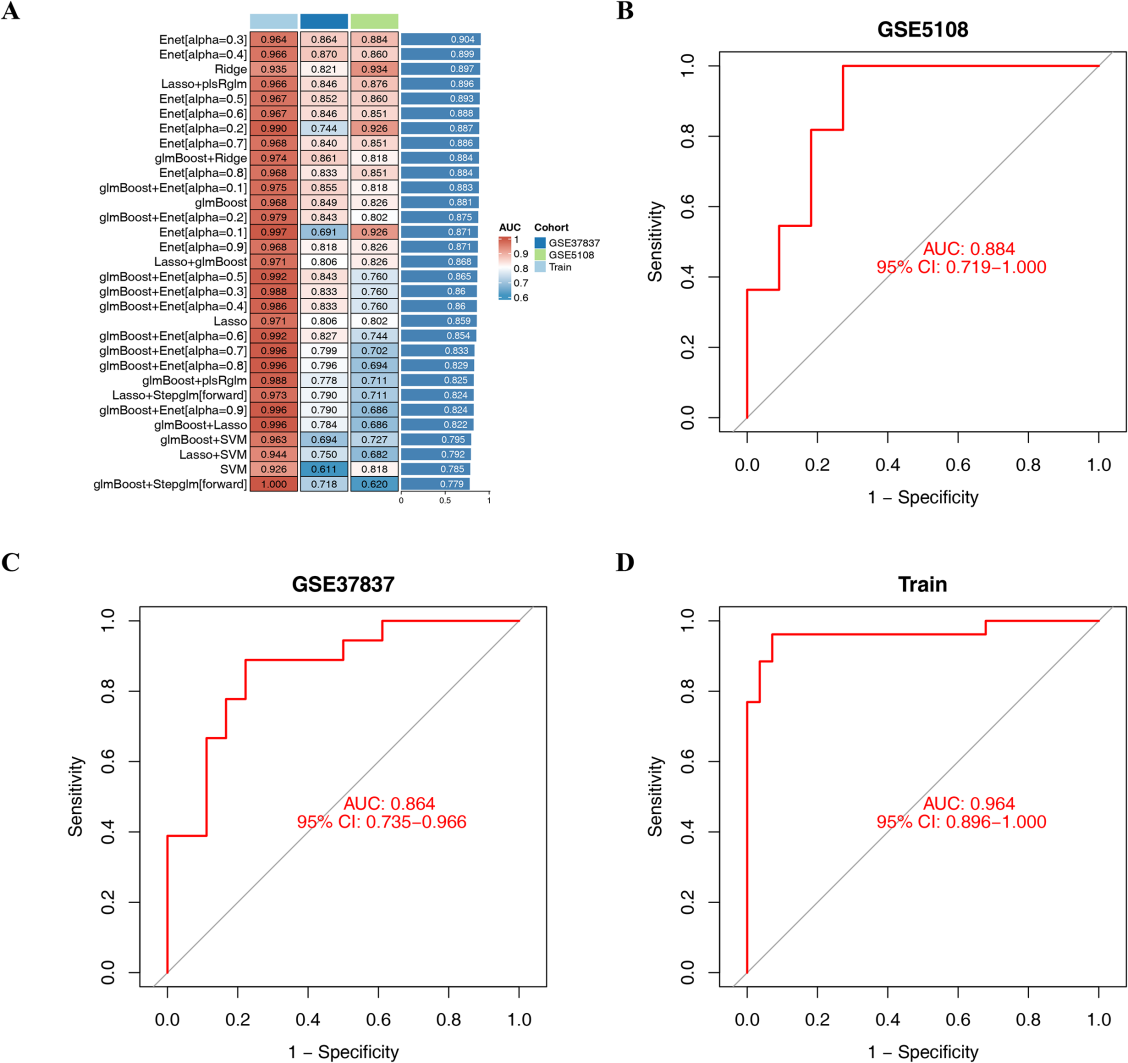
Identification of hub genes by integrated machine learning algorithms. **(A)** Concordance indices of 101 combinations constructed by 10 machine learning algorithms in the GEO dataset; ROC analysis results for the identified hub genes in GSE5108 **(B)**, GSE37837 **(C)**, and train **(D)**.

An analysis was conducted for common genes in the 1,404 DEGs, 489 brown module genes, and 201 candidate hub genes related to EMS were identified (**Figure 2A**). These candidate hub genes were enriched in 790 GO terms (647 BP, 75 CC, and 68 MF). The BP analysis underscored the predominant enrichment of hub genes in the processes, which included nuclear division, chromosome segregation, organelle fission, and nuclear chromosome segregation. The CC analysis unveiled the marked enrichment of hub genes within realms, which included the spindle, chromosomal region, and condensed chromosome. The MF analysis revealed that the hub genes were enriched in several activities, which included microtubule binding, tubulin binding, and microtubule motor activity, among others (**Figures 2B, C**; **Supplementary File 2**). The candidate genes were enriched in 27 KEGG pathways, which included the cell cycle, oocyte meiosis, p53 signaling pathway, DNA replication, and progesterone-mediated oocyte maturation, among others (**Figure 3D**). Enrichment analysis of 201 up- and down-regulated genes showed that 169 down-regulated genes were enriched in 505 BP results, 55 CC results, 55 MF results, and 20 KEGG pathways (**Figure 2E**). The significantly enriched terms were ranked by gen count, and the pathways with the same count, those with smaller p-values were prioritized. The top five enriched pathways were selected for visual display. Similarly, 32 up-regulated genes were enriched in 476 BP terms, 43 CC terms, 49 MF terms, and 6 KEGG pathways, and the top five were also selected for visual display (**Figure 2F**). The detailed enrichment pathway information is provided in **Supplementary File 3**. Further analysis shows that, LRP1 was enriched in 21 BP results and 7 MF results, which included regulation of binding negative regulation of peptidase activity, regulation of protein catabolic process, negative regulation of proteolysis, positive regulation of protein localization to membrane, regulation of phospholipase A2 activity, positive regulation of cholesterol efflux and scavenger receptor activity, etc, which was closely related to the process of EMS. And the detailed enrichment pathway information is provided in **Supplementary File 4**.

### 3.4 Machine learning-based identification of hub genes

A total of 201 candidate hub genes were subjected to a comprehensive analysis using the aforementioned machine learning pipeline integrating the ten algorithms to identify the optimal machine learning combination. According to the C-index of the training and testing cohorts, the Enet model (alpha = 0.3) yielded the highest C-index with a mean value of 0.904 and was thus identified as the optimal model, which comprised 30 hub genes (**Figure 3A**, **Supplementary File 5**). The ROC analysis results for the identified hub genes revealed that the AUC for the ROC curve was 0.884, 0.864, and 0.964 for GSE5108, GSE37837, and the Train set, respectively, indicating good discriminative performance of the model (**Figures 3B–D**).

### 3.5 Validation of diagnostic models and functional characterization of hub genes

**Figure 4:**
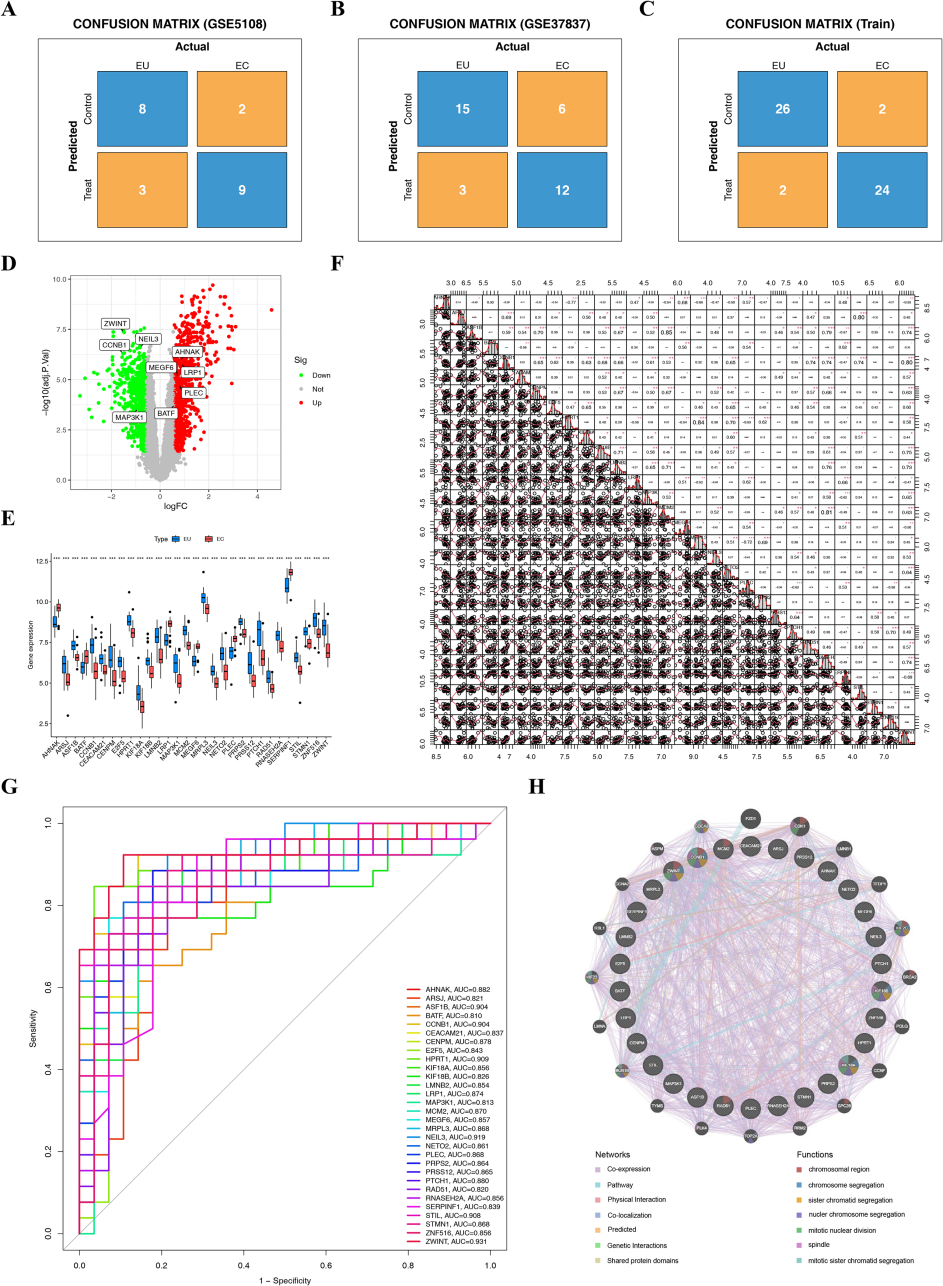
The diagnostic model based on 30 hub genes. Confusion matrix for GSE5108 **(A)**, GSE37837 **(B)**, and Train set **(C)**; **(D)** Volcanic diagram for the expression of each hub gene. The red and green nodes indicate the up- and down-DEGs, respectively; **(E)** The expression of hub genes in the EU and EC groups; **(F)** The correlation between each hub gene; **(G)** The ROC curve for each hub gene; **(H)** The interaction analysis of hub genes, and the construction of the gene co-expression network; Control: EU, Treat: EC; ^*^p < 0.05, ^**^p < 0.01, ^***^p < 0.001..

The two GEO and Train datasets were used to validate the performance of the model. The confusion matrix revealed that the model achieved similar accuracy (GSE5108: 0.77; GSE37837: 0.75; Train: 0.93), precision (GSE5108: 0.75; GSE37837: 0.80; Train: 0.92), recall (GSE5108: 0.82; GSE37837: 0.67; Train: 0.92), and F1-score (GSE5108: 0.78; GSE37837: 0.73; Train: 0.92) among the datasets (**Figures 4A–C**), underscoring the good discrimination performance of the diagnostic model. The expression of the hub genes in each group is presented in **Figures 4D, E**. The correlation of each hub gene had a positive correlation between AHNAK vs. MEGF6, ARSJ vs. STIL, LRP1 vs. ERPINF, and MCM2 vs. NASEH, among others (**Figure 4F**). The ROC curve of these hub genes revealed that the AUC for all hub genes was >0.8, indicating that these genes have good diagnostic abilities for distinguishing EC from EU tissues (**Figure 4G**). Genes with similar functions to hub genes were predicted using GeneMANIA, and 20 genes were ultimately obtained. The predicted and hub genes are located in the outer and inner circles, respectively. Genetic interactions among hub genes, as well as predicted genes, were observed. These hub genes are involved in numerous functions. For instance, KIF18A is involved in mitotic sister chromatid segregation, while ZWINT is involved in the chromosomal region and chromosome segregation (**Figure 4H**).

### 3.6 LRP1-related differential genes and functional enrichment analysis results

**Figure 5:**
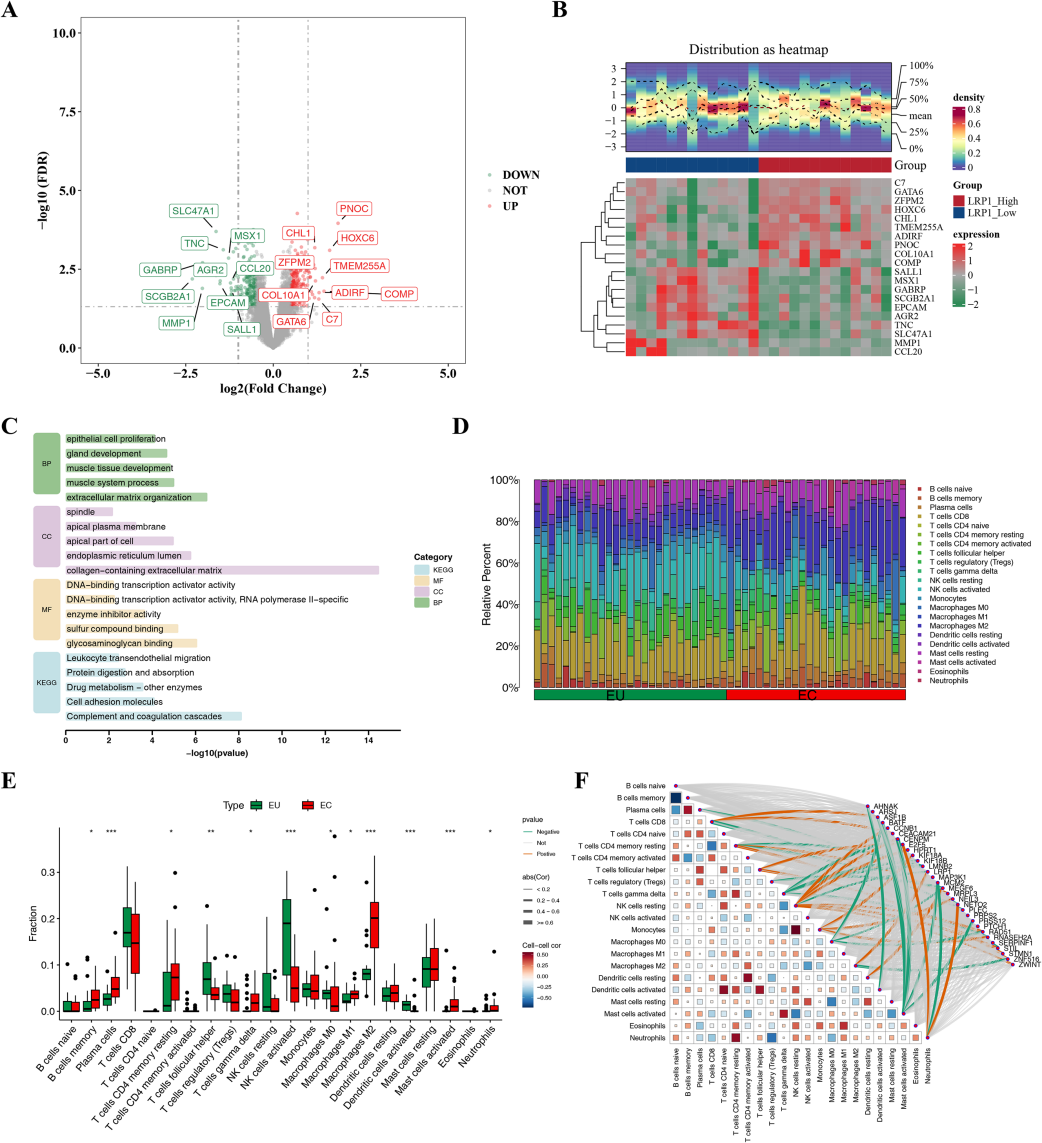
Differential Expression and Functional Enrichment Analysis of LRP1-Associated Genes. Volcano **(A)** and heat maps **(B)** of DEGs associated with LRP1; **(C)** Enrichment results of LRP1-related DEGs; **(D)** The proportion of 22 types of immune cells in each sample; **(E)** The infiltration rate for the 22 types of immune cells in the two groups; **(F)** The correlation analysis for the 22 types of immune cells with the hub genes; ^*^p < 0.05, ^**^p < 0.01, ^***^p < 0.001.

Although both LRP1 and AHNAK showed strong modulation in terms of logFC, LRP1 ranked highly across multiple analytic layers, including network centrality in the PPI/WGCNA framework, machine-learning feature importance, Mendelian randomization, and CellChat analysis, all of which linked LRP1 to EMS risk. In addition, functional enrichment and immune-correlation analyses indicated that LRP1 lies at the intersection of immune regulation, extracellular matrix remodeling, and intercellular signaling pathways highly relevant to EMS biology. Prior literature further supports a mechanistic link between LRP1 and inflammatory and stromal processes, providing a strong biological foundation for experimental follow-up. By contrast, although AHNAK showed differential expression, it did not demonstrate the same level of convergence across causal inference, immune association, and pathway analyses. Therefore, we prioritized LRP1 as the most integrative and mechanistically informative candidate for downstream validation. Initially, we identified LRP1-related differential genes and analyzed functions involved in DEGs in the GSE7305, GSE11691, and GSE25628 datasets. Following this, the possible biological pathways in which LRP1 might be involved within the EMS were investigated. Differential gene expression analysis identified a total of 425 DEGs between the LRP1_High and LRP1_Low groups, including 205 downregulated and 220 upregulated genes. A volcano plot generated using the ggplot2 R package (3.5.2) (**Figure 5A**) highlighted the top 10 upregulated and downregulated genes based on |log_2_FC|. Additionally, a heatmap created with the ComplexHeatmap package (2.16.0) displayed the expression patterns of the top 10 downregulated and top 10 upregulated genes (**Figure 5B**). Functional enrichment analysis of these DEGs yielded 813 BP terms, such as gland development, 70 CC terms, such as apical plasma membrane, 85 MF terms, such as DNA-binding transcription activator activity, and 12 KEGG pathways, such as protein digestion and absorption. Besides, the top five most significant pathways were visualized (**Figure 5C**). These pathways all revolved around cellular life activities, and DEGs were significantly enriched in these functions and pathways, indicating that LRP1 played a core role in regulating key biological processes such as cell growth and development, substance metabolism, signal transduction, and interaction with the outside world. In conclusion, the above results revealed the biological processes and signaling pathways potentially modulated by LRP1 expression differences and provided insights into the mechanisms underlying LRP1’s role in EMS.

### 3.7 Correlation analysis of immune cell infiltration characteristics and LRP1 in endometriosis

Accumulated evidence has demonstrated that EMS is closely associated with inflammation and immune processes (38). In this regard, the CIBERSORT algorithm was used to infer the immune cell abundances and characteristics and investigate the relationship between hub genes and immune cell infiltration. The proportion of the 22 types of immune cells in each sample is presented in **Figure 5D**. Compared to the EU endometrium, the EC endometrium had a higher infiltration rate of multiple types of immune cells, which included B-cell memory, plasma cells, T cells CD4 memory resting, T cells gamma delta, macrophages (M0, M1, and M2), mast cells activated, and neutrophils. On the other hand, the EC endometrium had a lower infiltration rate of T follicular helper cells, NK cells activated, and dendritic cells activated (**Figure 5E**). The correlation analysis for the 22 types of immune cells revealed that AHNAK was negatively correlated to dendritic cell activation, and ASF1B was positively correlated to T cell CD8, but negatively correlated to activated mast cells. Furthermore, NETO2 was positively correlated with neutrophils, but negatively correlated with T cell CD4 memory restoration. Notably, LRP1 was positively correlated to M2 macrophages, but negatively correlated to NK cell resting, among others (**Figure 5F**).

### 3.8 Mendelian randomization confirmed a causal association between LRP1 and EMS risk

**Figure 6:**
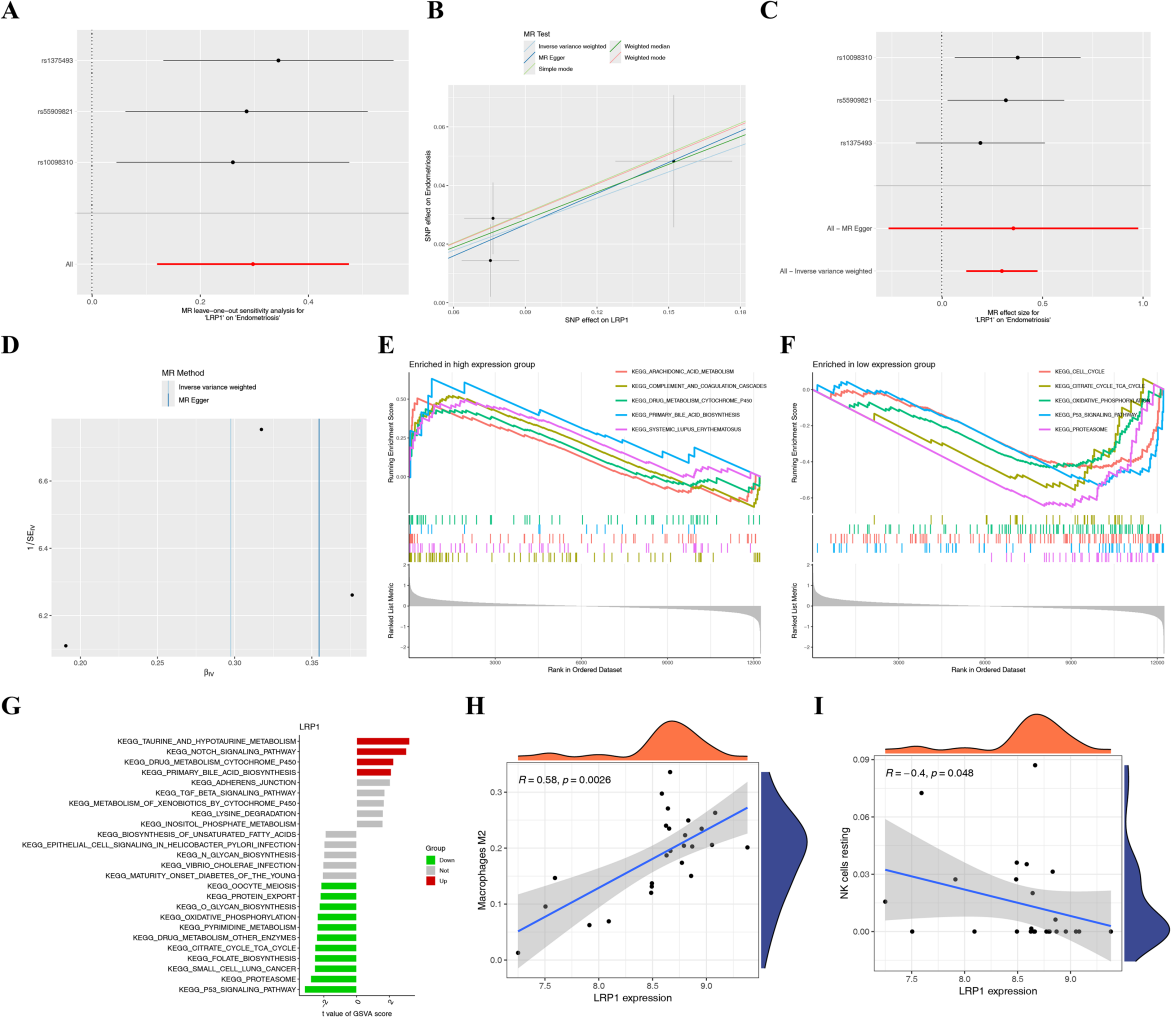
**(A-D)** The Mendelian randomization analysis results. **(A)** Scatter plot for the causal effects of LRP1 on the risk of EMS; **(B)** Forest plot for each SNP’s causal impact on EMS risk; **(C)** Funnel plot of overall heterogeneity in the MR estimation of the impact of LRP1 on EMS; **(D)** The result chart for the leave-one method revealed that when one SNP was removed, there was no significant difference in the causal effect of LRP1 on risk of EMS; **(E-I)** The GSEA, GSVA, and immuneCor analysis results for LRP1 in combined dataset (GSE7305, GSE11691 and GSE25628); **(E)** The top five active biological functions in the group with high LRP1 expression; **(F)** The top five active biological functions in the group with low LRP1 expression; **(G)** GSVA analysis for the high expression of LRP1; **(H, I)** ImmuneCor analysis for LRP1 associated to M2 macrophages and NK cell resting.

Using the MR analysis method, the causal relationship between LRP1 and EMS was investigated. In the MR analysis, SNPs related to confounding factors and outcomes, as well as incompatible alleles or those that exhibited moderate allele frequency palindromic patterns, were excluded. Then, 12 EMS-related SNPs that met the three key hypotheses of the MR analysis were retained for further analysis. The comprehensive details of IVs for EMS in the MR analysis are presented in **Supplementary File 6**. The IVW analysis revealed a positive association between LRP1 SNPs (rs10098310, rs1375493, and rs55909821) and EMS risk (OR: 1.3464, 95% CI: 1.1276-1.6077, *p* = 0.0010) (**Figure 6A**, **Supplementary File 7**). The presence of horizontal pleiotropy was examined using MR Egger regression analysis, and the results revealed that pleiotropy was unlikely to bias the causal relationships (*p*>0.05; **Figure 6B**, **Supplementary File 8**). The causal effects of the funnel plot were roughly symmetrical, and no horizontal pleiotropy was observed in the intercept of the MR Egger regression (*p* = 0.420), further indicating that pleiotropy does not bias towards the causal effects (**Figure 6C**). Next, systematic MR analysis was conducted on the remaining SNPs after removing each SNP (**Figure 6D**). The consistency of the results indicated a significant causal relationship between the calculated results of all SNPs. This shows that no dominant SNPs at the LRP1 level were linked to EMS, further validating the validity of the MR results.

### 3.9 Association analysis between LRP1 expression and EMS-related signaling pathways

**Figure 7:**
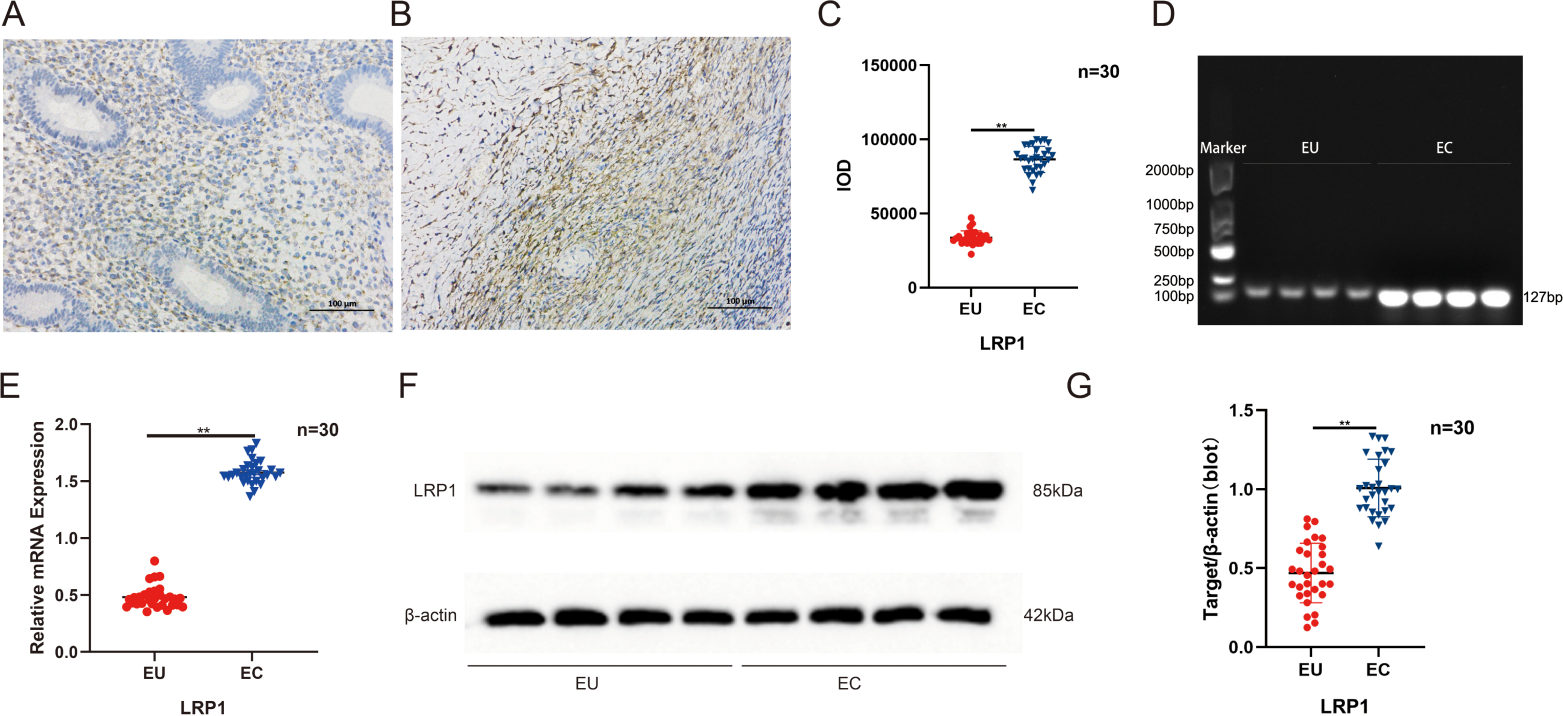
Localization and expression verification of LRP1 in EU and EC tissues. **(A)** IHC results for the LRP1 localization in EU tissues; **(B)** IHC result for the LRP1 localization in EC tissues; **(C)** Semi-quantitative analysis of LRP1 protein expression; **(D)** PCR results for LRP1; **(E)** The qRT-PCR results for LRP1; **(F)** Western blot results of LRP1; **(G)** The quantitative data from E, ^**^p < 0.01. IOD, Integrated Optical Density.

Based on LRP1, which had a significant causal relationship with EMS, the signaling pathways involved in EMS that were affected by LRP1 were investigated through GSEA and GSVA. Firstly, in the MR analysis, LRP1 was identified as the top gene associated with EMS. The GSEA analysis revealed that the elevated expression of LRP1 was mainly associated with the enriched signaling pathways, such as arachidonic acid metabolism, complement and coagulation cascades, drug metabolism cytochrome P450, primary bile acid biosynthesis, and systemic lupus erythematosus (**Figure 6E**), while the low expression of LRP1 was mainly associated with the involved signaling pathways, such as cell cycle, citrate cycle TCA cycle, oxidative phosphorylation, P53 signaling pathway, and proteasome (**Figure 6F**). The GSVA analysis results revealed that the high expression of LRP1 was linked to multiple signaling pathways, which included taurine and hypo-taurine metabolism, notch signaling pathway, drug metabolism cytochrome P450, and primary bile ACID biosynthesis, while the low expression of LRP1 was linked to other signaling pathways, such as oocyte meiosis, protein export, O-glycan biosynthesis, oxidative phosphorylation, pyrimidine metabolism, drug metabolism-other enzymes, citrate cycle, TCA cycle, folate biosynthesis, small cell lung cancer, proteasome, and the P53 signaling pathway (**Figure 6G**). The immuneCor analysis revealed that LRP1 was positively associated with M2 macrophages (**Figure 6H**) but negatively associated with resting NK cells (**Figure 7I**).

### 3.10 Verification of LRP1 upregulation in endometriosis tissues

To further validate the protein expression of LRP1 in EU and EC tissues, IHC was performed for the paraffin sections obtained from the 30 EU and 30 EC tissue samples. The IHC images revealed that LRP1 is expressed in both EU and EC tissues, and localized around blood vessels (**Figures 7A, B**), and that EC had higher LRP1 protein expression levels than EU (**Figure 7C**). Subsequently, the RNA expression levels were compared between the EC (n=30) and EU (n=30) samples by PCR (**Figure 7D**) and qRT-PCR (**Figure 7E**) analyses. The PCR analysis results revealed that the LRP1 RNA expression was upregulated in EC samples, when compared to EU samples (**Figure 7D**). Furthermore, the qRT-PCR results revealed that the RNA expression of LRP1 was significantly higher in EC samples, when compared to EU samples (**Figure 7E**). In order to validate and compare the protein levels of LRP1 between the EC and EU samples, the same EC (n=30) and EU (n=30) samples used for the RNA analysis were analyzed. As shown in **Figures 7F, G**, the LRP1 protein levels were significantly upregulated in the EC samples, when compared to the EU samples, indicating that the abnormal upregulation of LRP1 protein levels in EC tissues may have been due to the changes in transcriptional level. Notably, the present results are consistent with the published microarray data that demonstrated the increase in expression of LRP1 in EC samples (31).

### 3.11 The scRNA-seq revealed the immune landscape and communication network of LRP1

**Figure 8:**
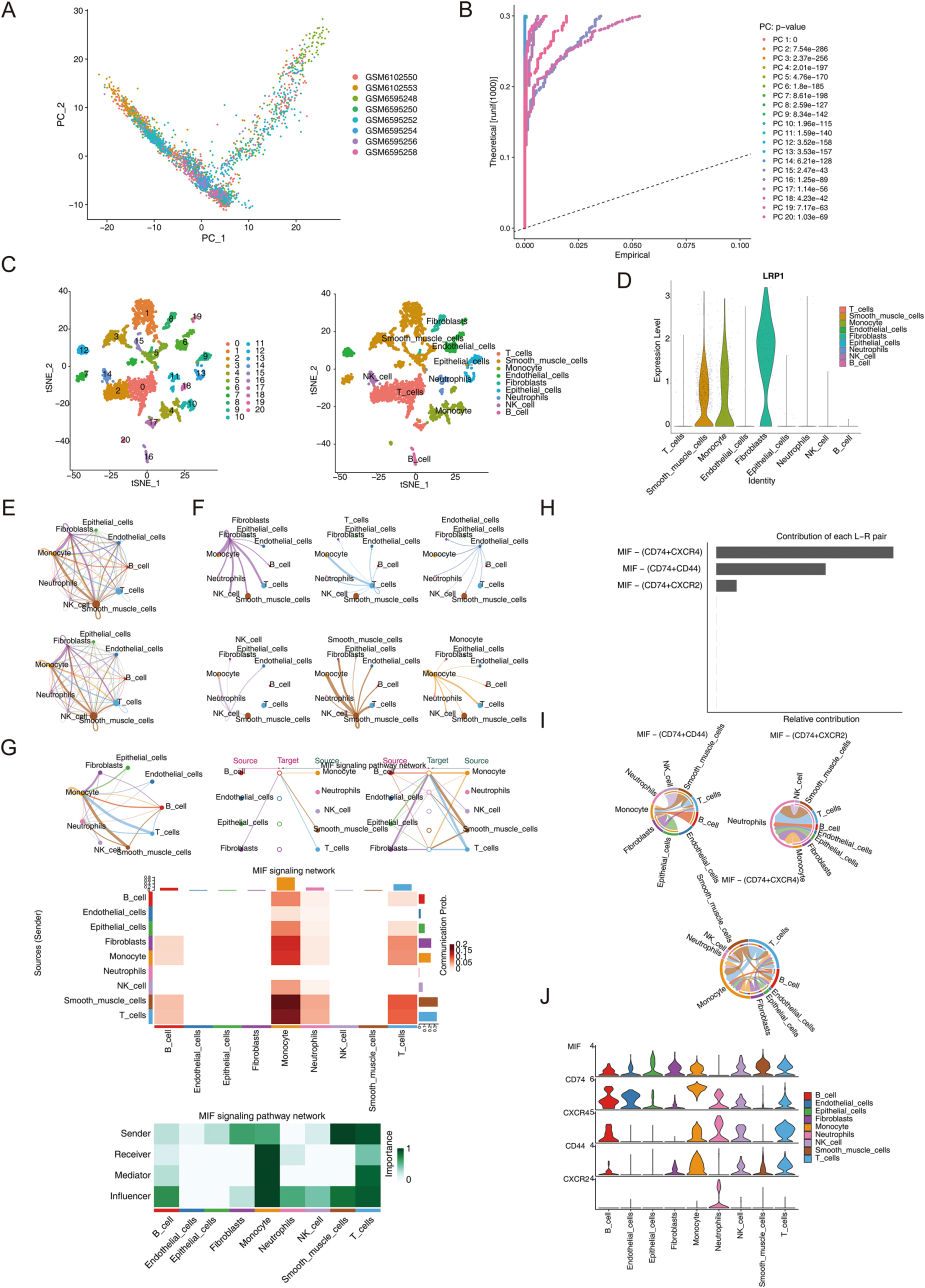
Cell communication network diagram. **(A)** Single-cell PCA analysis; **(B)** Single-cell JackStraw graph; **(C)** A total of 20 clusters was identified for all EC and EU samples after uniform manifold approximation and projection analysis. Nine cell subtypes were identified using the SingleR annotation method; **(D)** LRP1 was mainly expressed in fibroblasts, monocytes, and smooth muscle cells; **(E)** Receptor-ligand pair interactions between immune cells; **(F)** Cell–cell communication interaction in the MIF signaling pathway; **(G)** The interactions between immune cells, and the contribution weight of outgoing and incoming signaling patterns of target cell types related to the MIF signal pathway network; **(H)** Receptor-ligand pair interactions between immune cells; **(I)** Ligand-receptor interaction linked to the MIF signaling pathway. The CD74/CXCR4 ligand-receptor pair is the leading contributor, followed by the CD74/CD44 ligand and CD74/CXCR2 receptor pair; **(J)** The distribution and expression levels of signal genes related to the MIF signal pathway network.

To investigate the interaction patterns of immune cells during the progression of EMS, we processed and analyzed the scRNA-seq dataset. The scRNA-seq data obtained from GSE179640 in the GEO database were employed to analyze and identify the different types of immune cells in the EMS immune microenvironment, and the results were visualized using the dimensionality reduction algorithm (t-SNE; **Supplementary File 9**). After data standardization, PCA analysis was performed on the samples. Meanwhile, the JackStrawPlot function was used to assess the statistical significance of each principal component (PC) by comparing the distribution of p-values, aiding in the selection of PCs for further analysis (**Figures 8A, B**). Nine types of cells were identified (T cells, smooth muscle cells, monocytes, endothelial cells, fibroblasts, epithelial cells, neutrophils, NK cells, and B cells) from the single-cell transcriptomic data (**Figure 8C**). In the single-cell sequencing analysis, the GSE179640 dataset was used to conduct quality control and t-SNE dimensionality reduction on the single-cell transcriptome data from 8 EMS patients. A total of nine distinct cell types were identified. The expression of LRP1 showed significant cell-type specificity, with the highest expression levels in fibroblasts, followed by monocytes and smooth muscle cells. In contrast, the LRP1 expression levels in endothelial cells and epithelial cells are relatively low (**Figure 8D**). Next, an analysis was conducted on the dataset using CellChat to determine the enriched interactions between different types of cells. The results revealed that numerous types of cells, which included monocytes, fibroblasts, neutrophils, NK cells, epithelial cells, endothelial cells, and other cell populations, closely interacted in the EMS environment (**Figure 8E**). The CellChat software package contains a receptor-ligand interaction database that includes 2,021 validated molecular interactions. The CellChat analysis results revealed that there were close interactions among epithelial cells, endothelial cells, B cells, T cells, smooth muscle cells, NK cells, neutrophils, monocytes, and fibroblasts (**Figure 8F**).

Next, CellChat was used to identify the interactions between immune cells in the EMS microenvironment. To explore the key cell - cell interactions in the EMS microenvironment, we dissected the receptor - ligand pairs associated with LRP1. LRP1 was highly expressed in monocytes, fibroblasts, and other cell types. These cells had specific signaling interactions with other cell populations within the “MIF signaling pathway network”, reflecting their involvement in intercellular communication. Ligand-receptor interaction analysis revealed that the MIF signaling pathway was of great importance in the cell communication network. These findings implied that LRP1 might participate in the pathological process of EMS by regulating the signal transduction of related cells in the MIF signaling pathway, thereby affecting immune cell interactions and the input - output signal dynamics of various cell types (**Figure 8G**). Next, the contribution of each ligand-receptor pair to the entire signaling pathway was evaluated. The present analysis results revealed that the CD74/CXCR4 ligand-receptor pair interaction played a dominant role in contributing to the entirely enriched signal pathway, followed by the CD74/CD44 ligand and CD74/CXCR2 receptor pair (**Figure 8H**). Subsequently, the interactions between these ligand-receptor pairs in 12 immune cells were dissected, and it was found that CD74 and CD44 might communicate via sending signals to smooth muscle cells, epithelial cells, NK cells, B cells, and fibroblasts, which may interact with monocytes and T cells. Furthermore, the CD74-CXCR2 ligand-receptor mainly worked via sending signals from NK cells, smooth muscle cells, T cells, B cells, endothelial cells, epithelial cells, monocytes, and fibroblasts, which may interact with neutrophils through the interaction. Moreover, the CD74-CXCR4 ligand-receptor communicated by sending signals from fibroblasts, epithelial cells, endothelial cells, NK cells, and smooth muscle cells, which may interact with monocytes, T cells, and B cells (**Figure 8I**). Violin plots were used to visualize the distribution of gene expression signals in the MIF signaling pathway inferred by CellChat. The analysis results revealed that CD74 was expressed in B cells, endothelial cells, epithelial cells, fibroblasts, monocytes, neutrophils, NK cells, smooth muscle cells, and T cells. Furthermore, its ligand CXCR4 was mainly expressed in B cells, monocytes, neutrophils, NK cells, and T cells. Moreover, the present analysis results revealed that CD44 was mainly expressed in B cells, fibroblasts, monocytes, NK cells, smooth muscle cells, and T cells, while CXCR2 was mainly expressed in neutrophils (**Figure 8J**). These results suggest that LRP1 may induce the onset of EMS by affecting the cellular communication between various types of cells linked to the MIF signaling pathway.

### 3.12 The differentiation trajectory of fibroblasts

**Figure 9:**
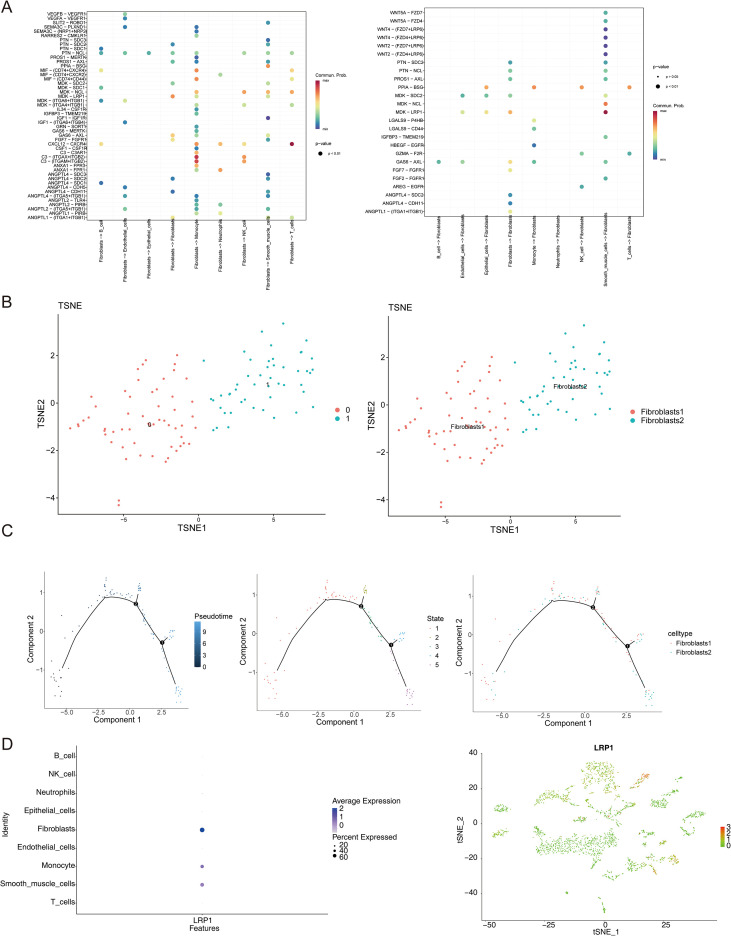
Characterization of fibroblast-associated signaling pathways, cell subpopulations, and differentiation trajectories in EMS. **(A)** Dot plot showing the intensities of ligand–receptor pairs involved in fibroblast-associated efferent and afferent signaling; **(B)** TSNE dimensionality reduction clustering analysis identifying key cell subpopulations; **(C)** Cell differentiation trajectory map; **(D)** Expression levels of core genes across different cell types.

The analysis showed that signal transduction among fibroblasts, monocytes, and smooth muscle cells involved multiple high-intensity ligand-receptor pairs (**Figure 9A**). To explore the fibroblasts’ differentiation trajectory and the expression level of LRP1 in cell differentiation, pseudotime analysis was performed. Fibroblasts were re-dimensionally reduced and clustered into two subtypes, fibroblasts1 and fibroblasts2 (**Figure 9B**). Pseudotime analysis demonstrated that the differentiation of fibroblasts ranged from dark blue to light blue, and the differentiation process was divided into 2 states and 2 cell subtypes (**Figure 9C**). Cell abundance analysis revealed the distribution of fibroblasts across different samples, with high expression of LRP1 observed in fibroblasts, monocytes, and smooth muscle cells (**Figure 9D**).

### 3.13 LRP1 may promote the progression of endometriosis by mediating cell migration, invasion, and proliferation through the MIF signaling pathway

**Figure 10:**
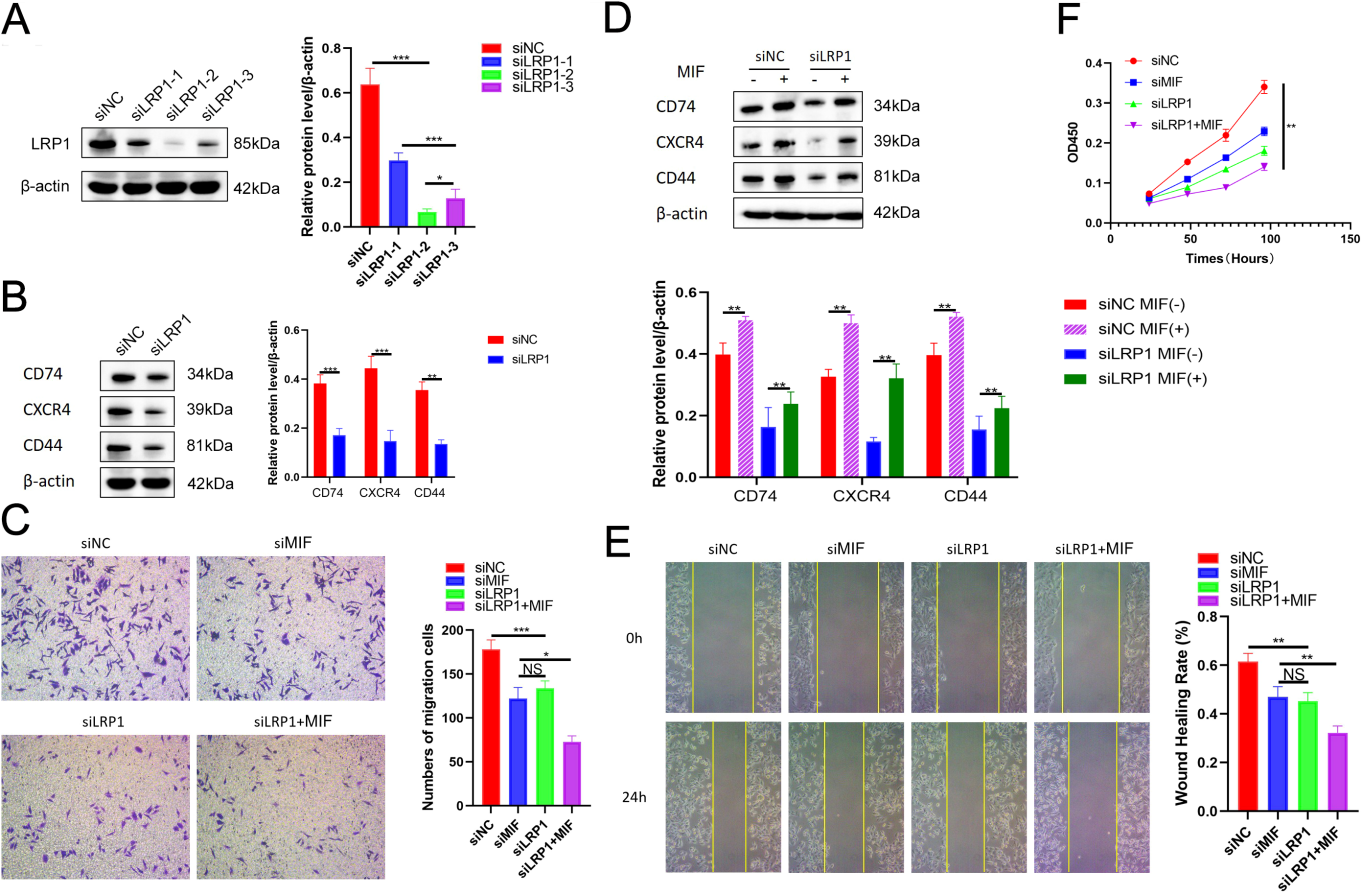
The reduction of LRP1 inhibits the EMS development by decreasing the proliferation, invasion, and migration, potentially through the MIF signaling pathway. **(A)** Western-blot of LRP1 level in si-LRP1; **(B)** The protein level of LRP1, CD74, CXCR4, and CD44 in cells transfected with si-NC and si-LRP1 with β-actin as the internal control; **(C)** Transwell assay showed the decreased cell invasion upon transfection with si-LRP1, si-MIF or si-MIF+LRP1 transfection; **(D)** Western-blot detection of CD74, CXCR4, and CD44 expression changes in response to MIF modulation under LRP1 silencing conditions; **(E)** Wound healing demonstrating decreased cell migration following si-LRP1, si-MIF or si-MIF+LRP1 transfection; **(F)** CCK8 assay showing the reduced cell viability in cells transfected with si-LRP1, si-MIF or si-MIF+LRP1. Data are presented as means ± SD, NS: *p* ≥ 0.05; *p < 0.05; **p < 0.01; ***p < 0.001.

To investigate the functional role of LRP1 in the pathogenesis of EMS, we conducted loss-of-function experiments in primary human endometrial stromal cells (hESCs) isolated from the eutopic endometrium. Three independent siRNA sequences targeting LRP1 were tested (siLRP1-1, siLRP1-2, siLRP1-3), among which siLRP1–2 achieved the highest knockdown efficiency and was selected for subsequent experiments (**Figure 10A**). These results confirm the efficient and specific silencing of LRP1 in our model system. Western blot analysis showed that LRP1 knockdown significantly reduced the expression of key proteins in the MIF signaling cascade (**Figure 10B**), including CD74, CXCR4, and CD44.

To confirm that the observed effects are specifically mediated through the MIF pathway, we directly knocked down MIF using siRNA (**Supplementary Figure 2**). Transwell experiments showed that transfection with si-LRP1, si-MIF, or the combined si MIF+LRP1 significantly reduced cell invasion ability. (**Figure 10C**). Western blot analysis confirmed that the expression of key proteins in the MIF signaling cascade was significantly lower than that of MIF (+) (**Figure 10D**). The wound healing experiment further showed that the migration ability of cells was reduced after transfection with si-LRP1, si-MIF, or si-MIF+LRP1 (**Figure 10E**). The wound healing rate was calculated as [(C0-C24)/C0] ×100%. C0 represents the wound size at 0 hour, and C24 represents the wound size at 24 hours. Similarly, the CCK8 assay showed a decrease in cell survival rate when transfected with si-LRP1, si-MIF, or si-MIF+LRP1 (**Figure 10F**).

## 4 Discussion

EMS has no specific clinical symptoms, making it difficult to diagnose and often leading to delayed detection and treatment (39, 40). Delayed diagnosis may aggravate disease progression and significantly impair patients’ quality of life. Therefore, identifying effective biomarkers with predictive and diagnostic value is essential for improving early detection and therapeutic intervention in EMS.

In the present study, we integrated transcriptomic datasets, bioinformatics analysis, RNA profiling, and experimental validation to identify key genes involved in EMS pathogenesis. A total of 30 hub genes were screened, and a diagnostic model was constructed that effectively distinguished ectopic endometrium (EC) from eutopic endometrium (EU) samples with high discriminatory performance. Among these genes, LRP1 was identified as a key hub gene and a potential biomarker associated with EMS development based on validation analysis and functional analysis.

Previous studies have suggested that lipid metabolism may contribute to the pathogenesis of EMS, and the low-density lipoprotein receptor signaling pathway has been implicated in disease progression (41–46). As a multifunctional receptor, LRP1 participates in numerous biological processes by binding cytoplasmic signaling adaptor proteins and interacting with multiple ligands, thereby regulating lipid metabolism, cell proliferation, cell migration, and extracellular matrix remodeling (11, 47, 48). In addition, LRP1 can be cleaved by matrix metalloproteinases to generate soluble fragments that modulate inflammatory signaling pathways, suggesting a critical regulatory role in various pathological conditions, including tumor progression (49–56). However, the specific biological role of LRP1 in EMS remains largely unclear.

Notably, functional enrichment analysis in this study revealed that LRP1-related upregulated genes in EMS were mainly enriched in cell cycle-related pathways, indicating enhanced proliferation of ectopic endometrial cells. In contrast, LRP1-related downregulated genes were primarily associated with wound healing, extracellular matrix organization, peptidase inhibition, and complement cascade pathways, suggesting impaired tissue repair and dysregulated immune surveillance in EMS. These findings imply that LRP1 may contribute to EMS development by coordinating multiple dysregulated pathways, promoting ectopic cell proliferation while simultaneously disrupting immune responses and extracellular matrix homeostasis.

Increasing evidence indicates that immune and inflammatory responses play crucial roles in the pathogenesis of EMS (57). Our immune infiltration analysis demonstrated a significant imbalance in immune cell populations within ectopic lesions, particularly in macrophage subtypes (M0, M1, and M2). Macrophages are known to participate in endometrial adhesion, colonization, and angiogenesis, and can dynamically polarize toward either pro-inflammatory M1 or anti-inflammatory M2 phenotypes depending on microenvironmental signals (58). Previous studies have shown that macrophages undergo gradual M1-to-M2 polarization during lesion progression and tissue remodeling, which is closely associated with inflammatory changes in EMS lesions and may represent a key driver of disease development (59).

Single-cell RNA sequencing (scRNA-seq) analysis further revealed that LRP1 may participate in regulating intercellular communication via the macrophage migration inhibitory factor (MIF) signaling pathway. MIF is a multifunctional cytokine secreted primarily by activated macrophages and T cells and plays key roles in angiogenesis, tumorigenesis, inflammation, and autoimmune diseases. Increasing evidence suggests that MIF is closely associated with the pathogenesis of endometriosis (60, 61). MIF can promote local estrogen synthesis by activating aromatase within endometriotic lesions, while estrogen can, in turn, induce MIF expression, forming a positive feedback regulatory loop (62). In addition, MIF can activate macrophages and stimulate angiogenesis (63). Activated macrophages subsequently secrete various pro-inflammatory mediators, including VEGF, TNF-α, and interleukins, which contribute to endothelial cell proliferation, neovascularization, and inflammatory responses in ectopic endometrial tissues (64). Moreover, MIF can inhibit the tumor suppressor protein p53, preventing apoptosis of genetically damaged cells and thereby facilitating lesion survival and progression (65). These findings suggest that LRP1 may influence EMS progression by regulating macrophage-mediated inflammatory signaling and the MIF pathway.

In addition to immune regulation, LRP1 also plays roles in extracellular matrix remodeling and metabolic regulation. By interacting with the urokinase-type plasminogen activator receptor (uPAR) and urokinase-type plasminogen activator (uPA), LRP1 can activate plasminogen and regulate fibronectin and collagen remodeling, thereby influencing extracellular matrix structure and tissue remodeling (66). As an endocytic receptor, LRP1 also contributes to cellular metabolic regulation by controlling extracellular matrix protein degradation and turnover.

Importantly, LRP1 has been reported to regulate receptor abundance on the cell surface and modulate signaling networks involved in intercellular communication. Through ligand–receptor interactions, LRP1 can influence signal transduction in various immune cells, including macrophages (67, 68). Several ligand complexes involving LRP1 may participate in EMS pathogenesis through different mechanisms. For instance, the LRP1–α2-macroglobulin complex contributes to inflammatory and immune responses in EMS (69, 70). The LRP1–thrombospondin-1 complex plays an essential role in angiogenesis and has been associated with infertility in EMS patients (71, 72). Furthermore, MMP9, which is significantly upregulated in EMS and widely recognized as a clinical biomarker, may promote lesion invasion through interactions with LRP1 and regulation of extracellular matrix remodeling (73, 74).

Despite these findings, several limitations should be acknowledged. First, functional gain- and loss-of-function experiments for LRP1 were not conducted in this study, and the regulatory mechanisms of LRP1-mediated intercellular communication require further experimental validation. Second, the single-cell datasets used in this study were limited by relatively small sample sizes and sequencing depth. In addition, the diagnostic model lacks external validation using independent large-scale cohorts. Future studies should expand sample sizes, integrate spatial transcriptomics with functional experiments, and explore the tissue-specific regulatory mechanisms of LRP1. Furthermore, constructing multi-biomarker diagnostic models combining LRP1 with existing clinical indicators may improve diagnostic accuracy and facilitate clinical translation.

In conclusion, by integrating transcriptomic data, bioinformatics analysis, and single-cell sequencing, we developed a diagnostic model with strong discriminative performance for EMS. LRP1 was identified as a key hub gene that may regulate the EMS immune microenvironment through ligand–receptor interactions and macrophage-related signaling pathways. These findings provide novel insights into the molecular mechanisms underlying EMS and highlight LRP1 as a promising biomarker and potential therapeutic target.

## Data Availability

The original contributions presented in the study are included in the article/**Supplementary Material**. Further inquiries can be directed to the corresponding author.
