# Supplementary figures and images for: LRP1 as a potential diagnostic and immunomodulatory target in endometriosis: evidence from multi-omics and single-cell analyses
Source: Front Immunol. 2026 Apr 15;17:1735221. doi: 10.3389/fimmu.2026.1735221 (PMC13124487; doi:10.3389/fimmu.2026.1735221)

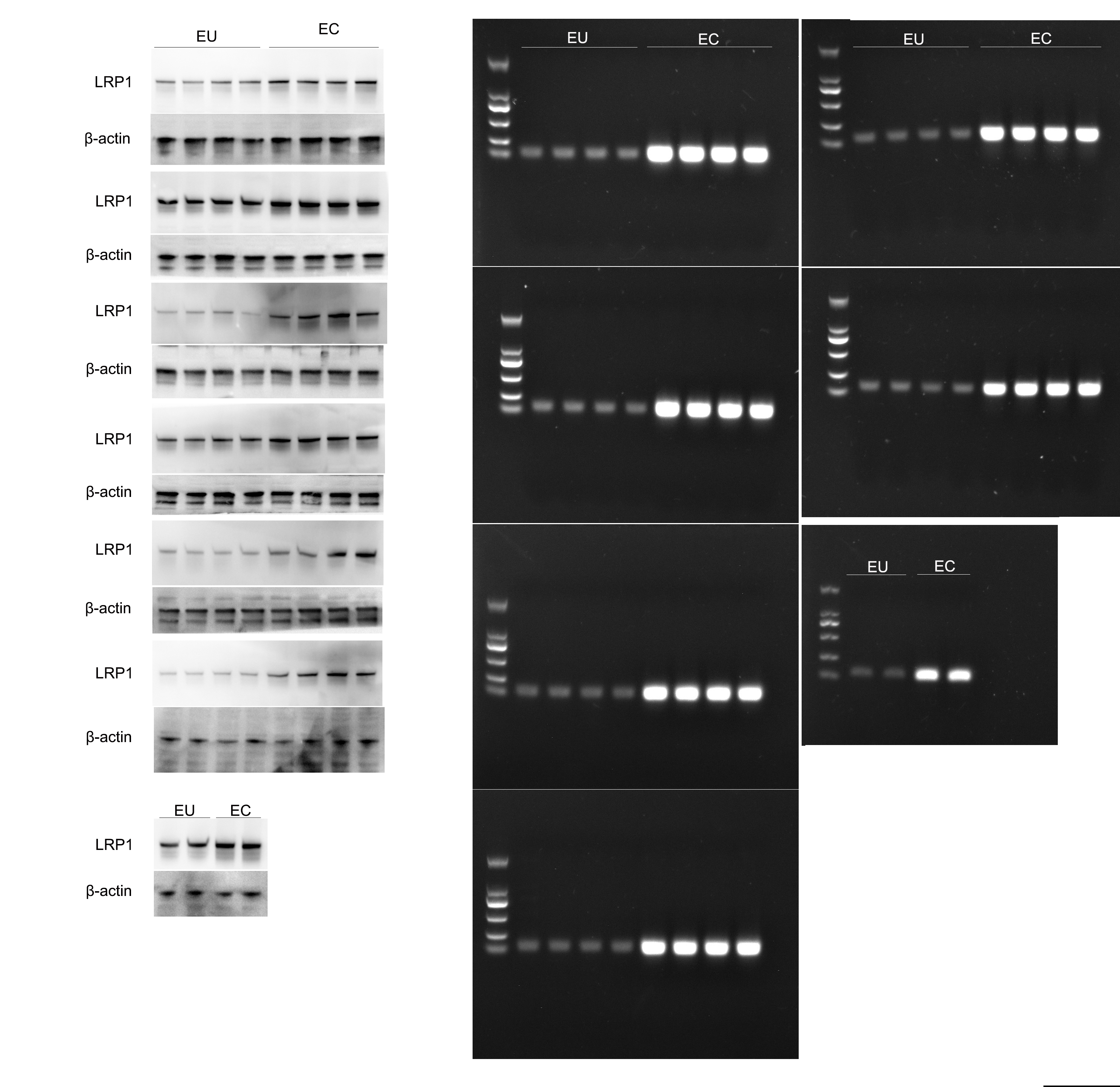

Supplement: Supplementary file 1 [file Image1.jpeg]

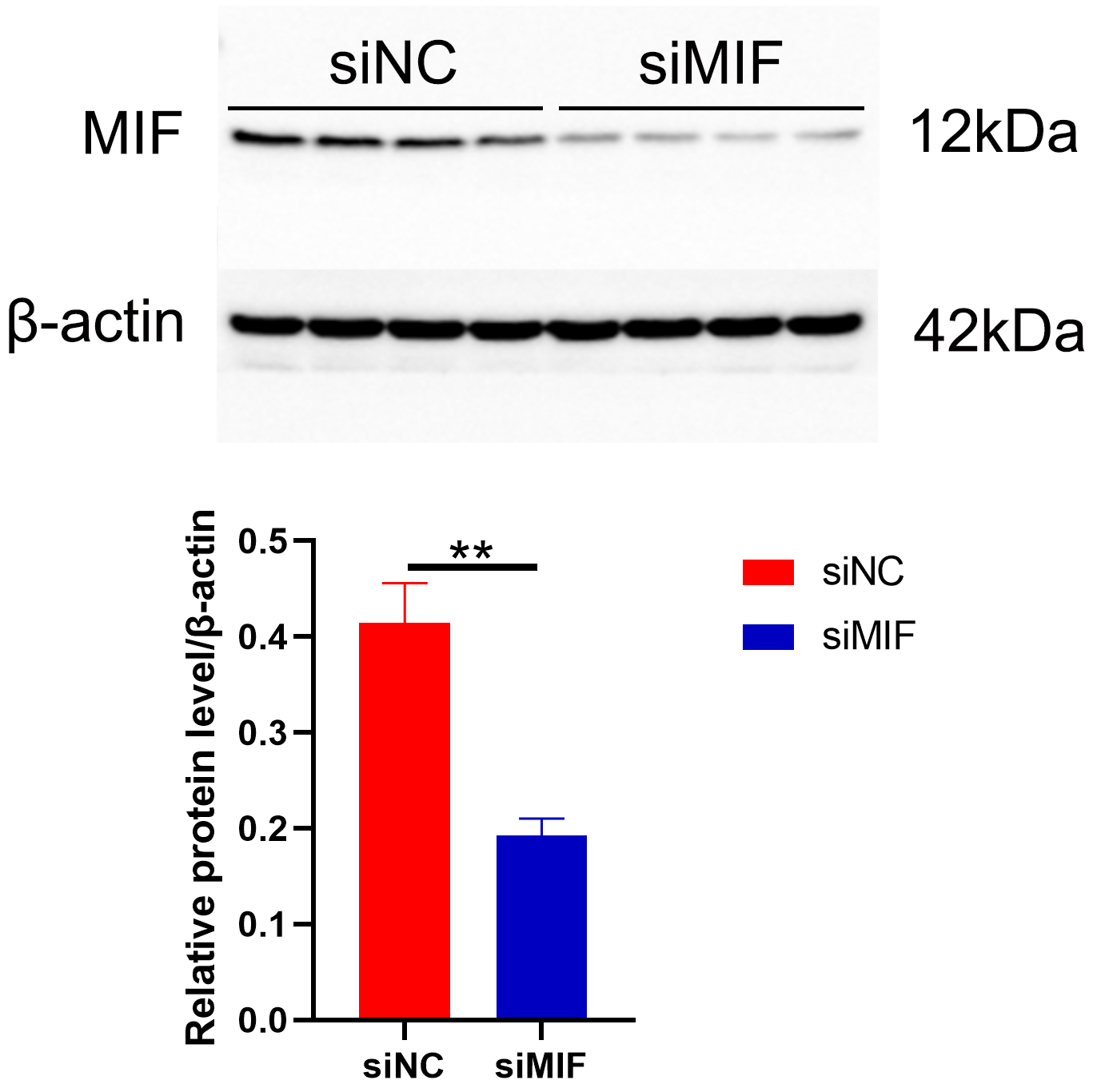

Supplement: Supplementary file 2 [file Image2.tif]
